# A Treatise on the Diseases of Infancy and Childhood

**Published:** 1891-01

**Authors:** 


A Treatise on the Diseases of Infancy and Childhood. By J. Lewis Smith, M. D., Clinical Professor of Diseases of Children, Bellevue Hospital Medical College; Professor to Charity Hospital, etc., etc. Seventh edition, thoroughly revised; with fifty-one illustrations. Philadelphia: Lea Brothers & Co. 1890.

It is unnecessary to make an extended review of a book so well known and highly appreciated as this, but it is a pleasure to announce a new edition. In every department it shows that it has been thoroughly revised, and that every advantage has been taken of recent advance in knowledge to bring it completely up to the times. The first part, devoted to the general hygienic care of the child from its birth to puberty, and the chapters on diathetic diseases, are deserving of careful study.

It would be well if every recent graduate, before he begins practice, could read the chapters on scarlet fever and take especial note of the directions for the proper method of isolation and disinfection.

What makes the work of Dr. Smith of especial value to young practitioners is the attention paid to diagnosis and the careful detail of treatment.

It is, undoubtedly, one of the best of treatises on children's diseases; and as a text-book for students and guide for young practitioners it is unsurpassed. DeL. R.

